# 3D scans, angles of repose and bulk densities of 108 bulk material heaps

**DOI:** 10.1038/sdata.2018.102

**Published:** 2018-05-29

**Authors:** Michael Rackl, Florian E. Grötsch

**Affiliations:** 1 Technical University of Munich, Chair for Materials Handling, Material Flow, Logistics, Boltzmannstraße 15, 85748 Garching, Germany

**Keywords:** Mechanical engineering, Characterization and analytical techniques

## Abstract

This paper presents a dataset of spatial data, angles of repose and bulk densities collected from 108 bulk material heaps. The investigated materials were lignite, wood chips, limestone, blast furnace coke, fresh and dried corn grains, milk powder as well as bituminous coal. Sample sizes range from 16 liters to approximately 220 liters, and each measurement was repeated four times to allow for variance assessment. This dataset is particularly useful for researchers and engineers, who want to investigate the shape of bulk solid heaps, or who want to test or benchmark measurement methods concerning heaps of granular matter, such as the angle of repose.

## Background & Summary

**Figure 1:**
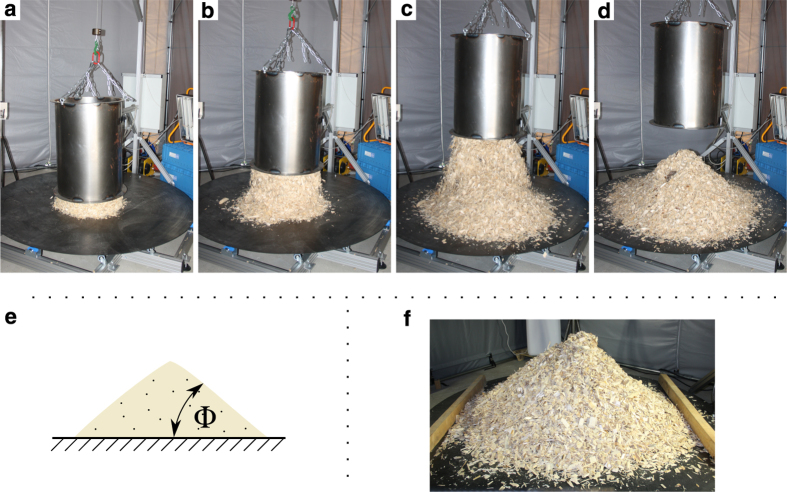
Experimental procedure for bulk solids. (**a**–**d**) Experimental formation of a wood chip heap and (bottom row) depiction of the angle of repose, Φ, (**e**) and an exemplary bulk solid heap of wood chips (**f**) with wooden battens aligned in parallel to the left and right of the heap.

Heaps are of great interest for bulk solids characterization. For instance, the angle of repose, a quantity which is widely reported in bulk material-related research, can be measured from heaps formed under gravity. Figure 1a–d shows a photo series of the formation of such a bulk solid heap, where a bottomless cylinder is filled with the bulk solid of interest and emptied while being lifted up. The angle of repose is defined as the angle between the horizontal (ground) and lateral surface lines of a conical heap (cf. Fig. 1e). Its theoretical foundation is based on continuum mechanics, which assumes a narrow particle size distribution and an ideal Coulomb material (ref. 1, p. 55). Although it is only applicable to cohesionless bulk solids in theory, it is also frequently reported for cohesive materials in practice^2–4^.

While the research community has agreed upon the theoretical basis for the angle of repose, a best practice for its measurement has not yet become apparent. Besides a few standards that describe ways to generate appropriate heaps for angle of repose measurement^5,6^, various other techniques are in use and there are two conventional ways of measuring its value: (i) direct measurement and (ii) indirect measurement. Using direct measurement, the angle of repose is obtained by holding a protractor to the surface line of the bulk solid heap. When using the indirect measurement method, the height, *h*, and diameter, *d*, of the heap are measured and the angle of repose is calculated from arctan (2*h/d*).

Both methods yield similar angles of repose, when the heap shape is conical. However, significant differences arise if the heap's shape is not conical. Typical (global) shape deviations include the convex or concave heap shape or a rounded tip^7^. Furthermore, recent research based on the data of the present data descriptor investigated the influence of local shape deviations^8,9^.

Either shape deviation can result from for instance cohesion, different particle shapes or segregation. If, for example, a global convex or concave shape deviation is present in a heap, a direct angle of repose measurement becomes ambiguous. In addition, heaps may not always be rotationally symmetric and different angles of repose are obtained when the measurement is repeated at various locations around the heap.

Due to the drawbacks of conventional measurement methods and increasing availability of computational power, researchers and engineers have adopted digital image processing methods to measure the angle of repose^7,10–12^. Using intricate image processing algorithms significantly increases the repeatability of angle of repose measurements. However, due to the lack of standardization, it also leads to a broader variety of measurement approaches. New measurement approaches for the angle of repose are almost always based on experiments the respective research groups carried out for their own studies^3,7,11,13^, which does not allow for direct comparison of these methods with regard to a particular heap.

In order to address the lack of a common database for bulk solid heaps, this data descriptor contains three-dimensional surface scans of 108 experimentally generated heaps of eight distinct bulk solids and three different sample volumes. The investigated bulk solids cover a wide range of particle sizes and industrial sectors and are backed by conventional, indirect angle of repose measurement data, determined according to the standard FEM 2.582 (ref. 14). The dataset is intended to provide researchers and engineers with bulk solid heap surface data for testing newly developed analysis methods and to provide a consistent basis for comparison and benchmarking of different analysis methods, for example for the angle of repose or related properties. Furthermore, single raw image data of the three-dimensional scans are provided. These can be useful to test spatial image reconstruction algorithms.

## Methods

### Investigated Bulk Solids

**Figure 2:**
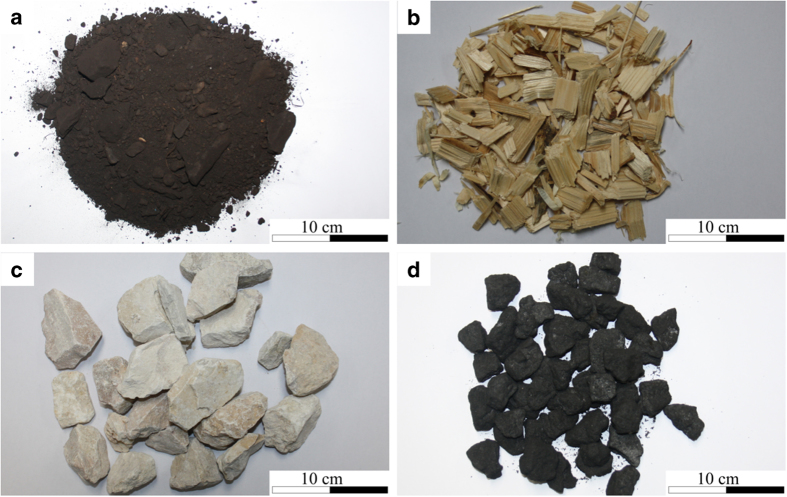
Sample photographs of the bulk solids I. (**a**) lignite, (**b**) wood chips, (**c**) limestone, (**d**) blast furnace coke (Figure from ref. 26).

Eight different bulk solids were investigated. Lignite, wood chips, limestone and blast furnace coke are depicted in Fig. 2. Lignite originated from the Hambach surface mining plant in Germany. The wood chips were classified as quality A-1 with particle size specification P45S and moisture class M15, according to the standard ISO 17255-4 (ref. 15). Lumped quicklime, produced from natural Jurassic limestone, were obtained in burned and crushed form and was graded 20 mm to 30 mm (ref. 16). Blast furnace coke was graded with particle sizes from 20 mm to 40 mm.

**Figure 3:**
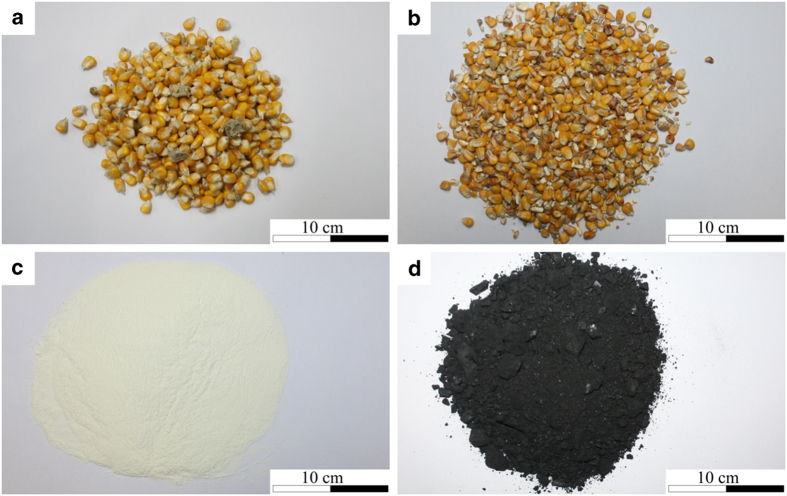
Sample photographs of the bulk solids II. (**a**) fresh corn grains, (**b**) dried corn grains, (**c**) milk powder, (**d**) bituminous coal (figure from ref. 26)

Figure 3 shows photographs of the four other bulk solids. Corn grains were purchased in freshly harvested and dried condition. The dried corn grains (purchased from BayWa AG, feedstock corn grains, batch 63) had been harvested in 2014 and the fresh corn grains were from the fall 2016 harvest in South Germany (Bavaria, Lower Bavaria; purchased from BayWa AG). The investigated milk powder was French spray-dried skimmed milk^17^, and the bituminous coal (steam coal) originated from Russia.

Where available, meta information for the bulk solids from technical data sheets or supplier specifications is provided in Data Citation 1.

## Experimental Procedure

### Conventional Measurements

**Figure 4:**
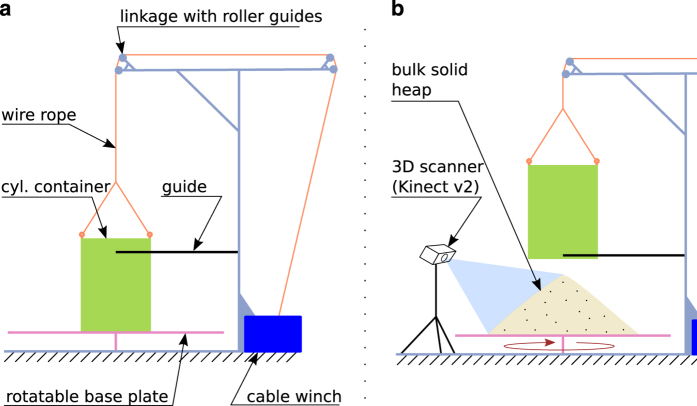
Experimental setup. Schematic drawing of the experimental apparatus (**a**) and setup for the three-dimensional surface scans of the heaps (**b**). Figures adapted from ref. 26.

The experimental apparatus and the measurement procedure were equivalent to those described in ref. 8. Figure 4 depicts the test stand, which consists of a rotatable base plate and an arrangement of levers to support and lift a topless and bottomless cylinder. Both the cylinder and the base plate were manufactured from rolled mild steel. The base plate is approximately 2m in diameter and the cylinder is available in three sizes, as listed in Table 1. An electrical lifting unit allows to lift a cylinder by means of a linkage with roller guides. During the lifting process, a lateral guide prevents the cylinder from swinging horizontally.

The experimental procedure was the same for each cylinder size. The respective cylinder was placed onto the base plate of the experimental apparatus and aligned flush with the guide. Depending on the sample volume of the bulk material, the material was poured into the cylinder using either buckets or troughs. The material contained in each respective bucket or trough was weighted using digital scales (Ref. 18, accuracy 0.02 kg) and the cylinder was filled to the prescribed filling height, which was marked inside the cylinder. While filling, the trough's/bucket's outer face was placed upon the cylinder's upper edge and the respective container slowly tilted. This ensured a consistent prescribed material drop height, *h*_*d*_, with regard to each cylinder size (Table 1). To check the filling height, the top material portion in the filled cylinder was carefully flattened by hand.

After the filling process, the cylinder was lifted vertically at a constant speed of either 33mms^−1^ (slow) or 142mms^−1^ (fast). As the lifting progressed, the bulk material inside the cylinder lost the latter's lateral support and formed a heap. After the cylinder was fully lifted, the heap was given time to settle until the particles on its surface and the base plate came to rest, which only took a few seconds. It shall be noted that, due to technical tolerances of the experimental apparatus, the cylinders could slightly tilt (max. 3°, w.r.t. vertical), at the moment when they were lifted off the base plate. This small deviation is unlikely to have had significant influence on the heap formation, however.

In the next step, the height of the bulk material heap, *H*, was measured with a custom-built marking gauge with a reading accuracy of 0.5cm.

As prescribed in the FEM 2.582 standard, two heap diameter measurements (*D*_*A*_, *D*_*B*_) were taken at crosswise alignment (perpendicularly) and their mean considered the heap diameter (cf. section Heap Diameter Measurement). The angles of repose were calculated according to equation 1 and rounded to a whole number as prescribed.

(1)α=arctan4HDA+DB

The bulk densities of the bulk materials, *ρ*_*B*_, were calculated from the known sample volume, *V*_*f*_, and the measured mass of the bulk material inside the cylinder, *m*_*f*_, equation 2.

(2)ρB=mfVf

### Three-dimensional Scans

The setup for three-dimensional scanning of the surfaces of the heaps is depicted in Fig. 4b. After a heap had settled, a Microsoft Kinect V2 3D scanner^19^ was arranged so that it pointed approximately perpendicularly to the lateral surface of the heap. The scanner then recorded depth images from all sides of the heap, while the bottom plate was turned incrementally. Note that the depth images were captured when the base plate was momentarily stopped.

The single depth images were reconstructed into one surface mesh, using the commercial software KScan 3D^20^. The program automatically arranges consecutive depth images to generate a three-dimensional surface mesh of the heap. Since the reconstruction algorithm can have a considerable influence on the final mesh, the original depth images are provided as PLY files (polygon file format a. k. a. Stanford Triangle format) for each heap, including the photographs from the 3D scanner. In these raw depth images, not only the test stand and the heap but also additional items, e. g., logs, can be seen. These items were sometimes needed as reference objects (so-called *landmarks*) to support the 3D reconstruction of highly rotationally symmetric heaps, for instance.

The reconstructed three-dimensional surface meshes were saved to STL file format (STereoLithography a.k.a. Standard Tessellation Language) and surface resolution was reduced to obtain a compromise between an exact representation of the heap surface and file size. Reference objects were manually removed and the meshes cleaned up. In addition, the coordinate system was translated and rotated so that the XY plane was approximately parallel to the base plate, and the Z axis passed roughly through what was considered the tip of the heap. The manually edited meshes are referred to as “processed” meshes, whereas the meshes are considered “raw” before manual cleanup.

### Experimental Plan

The experimental plan featured three factors: type of bulk solid (eight levels), cylinder size (three levels) and cylinder lifting speed (two levels). Lifting speed was initially kept constant at 142 mm s^−1^ and experiments were performed according to a full factorial design of experiments with each bulk solid and cylinder size. These 24 combinations were repeated four times (=96 experiments).

Based on an additional study plan, wood chips, dry corn grains and limestone were examined at a lifting speed of 33 mm s^−1^, in the medium-sized cylinder. This yielded three combinations, which were also repeated four times (=12 experiments). A total of 108 (96+12) experiments were conducted.

### Code availability

The provided KScan files were processed and saved with KScan3D version 1.2.02 (64 bit)^20^. STL files were handled and edited with Autodesk Meshmixer version 11.0.544 (ref. 21).

## Data Records

The datasets were made available from *figshare* (Data Citation 1).

## Meta Information

The file “additional bulk solids info.pdf” contains meta information with regard to the investigated bulk solids. The respective pieces of information were obtained from suppliers and technical data sheets, where available.

### Particle Size Distributions

In the comma-separated-value file “Particle_Size_Distribution.csv” (UTF-8 encoding), results from sieving analysis of the investigated bulk solids are stored. Sieving analysis is a basic measurement method to determine the particle size distribution of a bulk solid. These data were taken from ref. 8, where the exact same materials were examined.

### Conventional Measurements

The comma-separated-value file “BaseData.csv” (UTF-8 encoding) contains results from the conventional measurements, i. e., heap height, filling mass, heap diameters, angles of repose, measured mass and bulk densities. It also features a column with the median particle size of each bulk solid, which was interpolated from the data in “ParticleSizeDistributions.csv”.

### 3D Scanning

**Figure 5:**
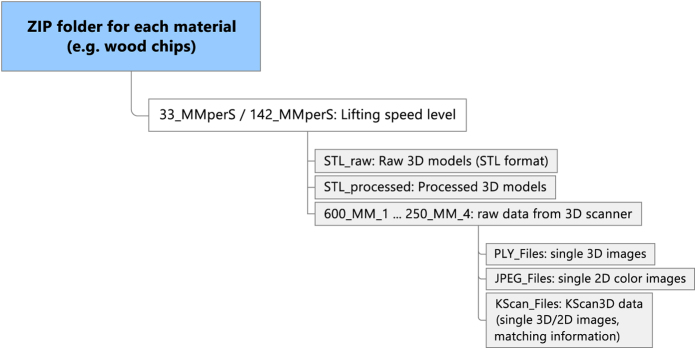
Folder structure of the ZIP files.

Data from the three-dimensional heap scans are stored in 8 separate ZIP archive files, one for each bulk material. Each archive has the same folder structure, presented in Fig. 5. The first level contains a maximum of two folders, one per lifting speed level. On the second level, there is one folder per cylinder diameter and measurement repetition. For example, the folder name “425_MM_2” denotes the second repetition with the 425 mm diameter cylinder. Each of these folders contains three subfolders called “JPG_Files”, “KScan_Files” and “PLY_Files”. “KScan_Files” holds the original files which were processed and saved with the software KScan3D. “JPG_Files” and “PLY_Files” contain the recorded 3D data from around the heap as single images (JPEG format) and scenes (PLY format), respectively. It should be noted that the filenames in these two folders correspond to each other, meaning that e. g., “425_MM_1_05.jpg” and “425_MM_1_05.ply” were recorded simultaneously by the 3D scanner and thus show the same perspective. Furthermore, on the second folder level, there are two folders named “STL_processed” and “STL_raw”, which include the processed and raw STL meshes (binary format) of each measurement repetition.

## Technical Validation

### Heap Diameter Measurement

**Figure 6:**
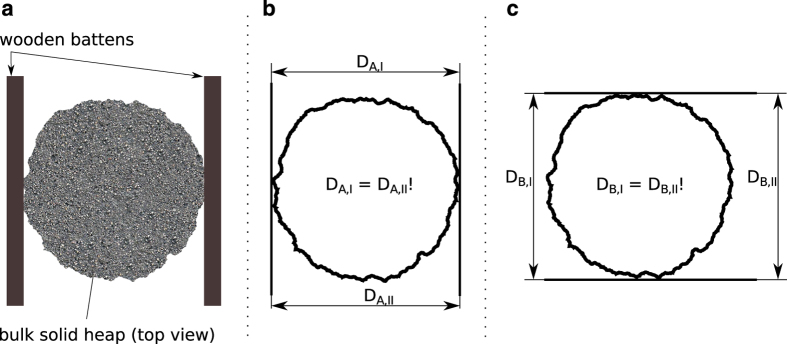
Heap diameter measurement. Indirect measurement with the help of wooden battens (**a**) and crosswise parallel alignment (**b**,**c**).

The heap diameter was difficult to measure directly due to its non-circular base shape and rather large dimensions of more than 1.5 m in the presented experiments. Thus, two straight wooden battens and a folding rule were used to measure the heap diameter. As shown in Fig. 6a, the wooden battens were tangentially aligned with the base area of the heap, and parallel to each other. The distance between them was measured at two locations as indicated in Fig. 6b and used to confirm parallel alignment (Fig. 6b), i. e., $D_{A}=D_{A,I}=D_{A,II}$. The same procedure was repeated to obtain *D*_*B*_ (Fig. 6c).

### 3D Scanning

Multiple studies have examined the accuracy of the Kinect v2's depth images^22–25^. Typical measurement errors range from a few to 30 mm. It should be noted that the measurement errors vary with regard to multiple factors, such as the distance of the scanned object to the 3D scanner as well as the surface condition of the object. For the distances at which the presented scans were recorded, a measurement error of approximately two to four millimeters was estimated from the studies mentioned above. Preliminary test runs with the presented 3D scanning setup confirmed this value range. Furthermore, the 3D scanner was given half an hour to reach a steady operating temperature, since it has been reported that Kinect v2's depth data are sensitive to the initial temperature increase^25^.

## Usage Notes

It can be helpful to convert STL meshes from binary to ASCII encoding, as some software does not support binary STL files.

## Additional information

**How to cite this article:** Rackl, M. & Grötsch, F. E. 3D scans, angles of repose and bulk densities of 108 bulk material heaps. *Sci. Data* 5:180102 doi: 10.1038/sdata.2018.102 (2018).

**Publisher’s note:** Springer Nature remains neutral with regard to jurisdictional claims in published maps and institutional affiliations.

## Supplementary Material



## Figures and Tables

**Table 1:** Inner diameters, *d*_*i*_, filling heights, h_*f*_, and resulting filled volumes, *V*_*f*_, of the cylindrical cylinders.

*d*_*i*_ (mm)	*h*_*f*_ (mm)	*V*_*f*_ (L)	*h*_*d*_ (mm)
250	325	16.0	350
425	545	77.3	600
600	780	221	800
